# PERMEPSY: a multicentre, randomized, double-blind proof-of-concept trial of personalized metacognitive training for adults with psychosis — a study protocol

**DOI:** 10.3389/fpsyt.2026.1711659

**Published:** 2026-03-06

**Authors:** Maria Lamarca, Claudia Requejo, Adrianna Aleksandrowicz, Adrien Goncalves, Martyna Kreżołek, Hanna Gelner, Justyna Piwińska, Rabea Fischer, Merle Schlechte, Alvaro Cavieres, Vanessa Acuña, Fabrice Berna, Steffen Moritz, Caroline König, Łukasz Gawęda, Susana Ochoa

**Affiliations:** 1 Research Unit, Parc Sanitari Sant Joan de Déu, Sant Boi de Llobregat, Barcelona, Spain; 2 Grup Etiopathogenesis and Treatment of Severe Mental Disorders (MERITT), Fundació Sant Joan de Déu, Institut de Recerca Sant Joan de Déu, Esplugues de Llobregat, Barcelona, Spain; 3 Consorcio de Investigación Biomédica en Red de Salud Mental (CIBERSAM), Instituto de Salud Carlos III, Madrid, Spain; 4 Departament de Psicologia Clínica i de la Salut, Facultat de Psicologia, Universitat Autònoma de Barcelona, Bellaterra, Cerdanyola del Vallès, Barcelona, Spain; 5 Facultad de Psicología, Universitat de Barcelona, Barcelona, Spain; 6 II Department of Psychiatry, Medical University of Warsaw, Warsaw, Poland; 7 University of Strasbourg, Inserm, Strasbourg, France; 8 Department of Psychiatry and Psychotherapy, University Medical Center Hamburg-Eppendorf, Hamburg, Germany; 9 Departamento de Psiquiatría, Escuela de Medicina, Facultad de Medicina, Universidad de Valparaíso, Valparaíso, Chile; 10 Unidad de Trastornos Psicóticos. Hospital Del Salvador de Valparaíso, Valparaíso, Chile; 11 Soft Computing Research Group at Intelligent Data Science and Artificial Intelligence Research Center Universitat Politècnica de Catalunya, Barcelona, Spain

**Keywords:** clinical trial, metacognitive training, PERMEPSY, personalized medicine, psychological interventions, psychosis, schizophrenia

## Abstract

**Background:**

While psychological interventions are effective at improving symptoms of psychosis, accessible, cost- and time-efficient treatments remain limited. Personalized medicine has emerged as a promising approach, tailoring interventions to individual needs. Metacognitive Training (MCT), with its established efficacy and adaptable format, is well-suited for personalization. The PERMEPSY project (Towards a Personalized Medicine Approach to Psychological Treatment for Psychosis) aims to deliver tailored MCT intervention for individuals with psychosis.

**Methods:**

PERMEPSY is an international study funded by ERAPerMed (JTC2022) involving five clinical partners (Spain, Chile, France, Germany, Poland) and one technological partner (Spain). The project involves a proof-of-concept clinical trial recruiting 51 participants from each center for a total of 255 adult participants with psychosis in a prospective study (Registration: NCT06603922, 19-09-2024). The trial will test the efficacy of a Machine Learning (ML)-derived platform at predicting clinical and functional outcomes from baseline scores and compare a personalized MCT (P-MCT) to a classical MCT based on the platform’s predictions.

**Aims:**

PERMEPSY seeks to (1) develop and test the predictive power of an algorithm that could support decision-making, and (2) ascertain whether P-MCT is more effective than MCT at improving key symptoms and cognitive impairments associated to psychosis.

**Results:**

A harmonized retrospective database enabled the development of a predictive ML algorithm, integrated into an innovative platform. This platform provides clinicians with the information needed to deliver P-MCT. Predictions include changes in positive symptoms (e.g., delusions), insight, self-esteem, and treatment adherence.

**Discussion:**

By integrating diverse data types and innovative technology, PERMEPSY addresses the need for personalized, effective treatment in psychosis, aiming to reduce individual and systemic burdens while supporting clinicians in their decision-making.

## 1 Introduction

Schizophrenia and other psychotic disorders are severe mental health disorders affecting approximately 2% of the global population (1), with a 12-month prevalence of 4.03 per 1000 persons (2) and an incidence of all psychotic disorders of 26.6 per 100.000 person-years (3), leading to significant levels of disability and generating a considerable burden for patients, caregivers, and healthcare systems. In Europe, the average annual direct cost per patient is estimated at 5.800€ (4).

Although pharmacological treatment with antipsychotics is widely used, it does not always improve functional outcomes, with discontinuation rates ranging from 50% to 75% among patients (5). In this context, psychological interventions have demonstrated to be beneficial, even in the absence of pharmacological treatment, with 40% of patients experiencing significant symptom improvement as a result of psychological interventions (6).

Metacognitive Training (MCT) is a low-threshold, manualized intervention available free of charge in 40 languages (available www.uke.de/mct). Based on the principles of Cognitive Behavioral Therapy (CBT), MCT is an effective psychological intervention for psychosis that has been incorporated into the latest World Health Organization (WHO) treatment guidelines (7), and is recommended by the German Psychiatric Association (DGPPN) (8), the German Psychological Society (DGP) (9), and the Australian and New Zealand College of Psychiatrists (10).Recent quantitative syntheses have provided a detailed picture of the specific clinical targets most responsive to MCT in schizophrenia. A comprehensive meta-review (11) reported medium to large effects on delusions (*g* = 0.64), positive symptoms (*g* = 0.47), and total psychotic symptoms (*g* = 0.39), with smaller but significant effects on hallucinations (*g* = 0.27) and negative symptoms (*g* = 0.23). Additional meta-analyses have shown improvements in general functioning (*g* = 0.41), self-esteem (*g* = 0.17), and cognitive biases (*g* = 0.16) (12), as well as global social cognition (*d* = 0.28) and theory of mind (*d* = 0.27) (13). MCT also improves insight (*g* = 0.35), although no significant effects have been found for global neurocognition (14), aligning with the intervention’s theoretical focus on metacognitive processes rather than fundamental cognitive capacities. These findings are consistent with a recent graded evidence review (15), which assigned a WFSBP-1 level of evidence to MCT for reducing positive symptoms, delusions, and total psychotic symptoms; and WFSBP-3 for hallucinations, negative symptoms, general functioning, theory of mind, jumping to conclusions, and insight. Together, this literature provides a detailed and clinically relevant framework of expected treatment targets. However, despite its effectiveness, there are currently no predictive models capable of assessing whether an individual is likely to respond well to MCT or which MCT modules would be most suitable for improving a patient’s prognosis.

Multiple studies (16–18) have identified cognitive biases, symptom severity, self-esteem, age of onset, and sex as key moderators of treatment efficacy. Similarly, gender differences in response to MCT have been established, with another study finding that women with psychosis respond differently and more favorably to MCT than men (19, 20).

Biomarkers are considered essential for identifying patient subgroups and enabling personalized approaches in precision medicine in psychiatry (21, 22). Considering biological variables, potential inflammatory biomarkers associated with MCT response include C-reactive protein, extensively studied in schizophrenia due to its correlation with symptom severity, and leukocyte counts, which are linked to grey matter volume (23, 24). Previous studies support the role of SP1 and SP4 transcription factors as biomarkers that may be modulated by MCT treatment (25–27). Therefore, considering the previous literature biomarkers may become an important moderator to consider in the treatment of psychosis, and enable subgroup analyses that could help determine biological profiles of treatment response.

The extensive research and quality of studies researching the effect of MCT provides an ideal context for developing a data-driven approach using an analytical pipeline based on machine learning (ML) based predictive models. Predictive models are used both for diagnosis and prognosis in the personalization of psychiatric healthcare (28, 29), and ML serves as a natural enabler of personalized medicine (30–32) when combined with knowledge extraction and explainability strategies (33).

Most researchers and clinicians agree that the treatment of psychotic patients should be personalized and tailored to their specific needs (34). However, its implementation in clinical practice remains limited and depends on overcoming several challenges One reason is the economic cost of personalization and its adoption by clinicians (35) among others. Another significant barrier is the limited scientific knowledge regarding the factors that should guide treatment customization, an ongoing challenge in mental health research (36).

The PERMEPSY Project emerges as a response to this need and available data, with the aim of developing a predictive prototype platform to assist clinicians in determining the optimal psychological MCT treatment strategy for each individual. Predictions are based on each individual’s personal characteristics and baseline scores in various clinical, cognitive and functional outcomes. These outcomes were selected both by data-driven insights derived from the ML analysis and an extensive review of the literature by consortium partners (11, 15, 19, 37, 38), which took place prior to the development of the prototype platform (39) to identify key indicators of treatment efficacy in psychosis. This innovative approach leverages ML techniques to analyze a broad spectrum of variables, from environmental factors to biomarkers, thus reflecting the multifactorial nature of mental illnesses.

The technological solutions employed in PERMEPSY, such as ML, can facilitate the identification of key variables in predicting treatment response. This may help clinicians focus on both objective and subjective problematic areas, consider the course of illness, and adopt a gender-sensitive approach early on in the treatment stage. The project also integrates methodological advancements, ensuring the personalized treatment for each patient is data-driven and supported by the prototype platform.

The overarching goal is to develop an open platform that supports clinicians in treatment decision-making, enabling them to predict MCT response, recommend personalized MCT (P-MCT) treatment, and provide evidence-based information. This will assist clinicians in making informed decisions regarding psychological interventions for psychosis. A clinical trial will be conducted to validate both the platform and the predictive model for personalized MCT.

Therefore, the primary objective of this prospective clinical trial is (1) to evaluate the efficacy of P-MCT compared to classical MCT in individuals diagnosed with psychosis, focusing on improvements in positive symptoms (especially delusions), insight, self-esteem, and treatment adherence. Moreover, some specific objectives of this trial include: (2) to validate the ML-based predictive model’s capacity to anticipate MCT treatment response, (3) to evaluate patient satisfaction with P-MCT comparing to classical MCT, integrating both quantitative measures and qualitative insights from focus groups, and (4) to refine the platform’s predictive algorithm through integration of treatment outcome data.

Given that P-MCT has not been previously evaluated and that the ML platform is a novel tool, hypotheses 2–4 are considered exploratory. Our hypotheses are as follows:

H1 - Primary (Comparative efficacy): P-MCT will yield comparable or superior improvements than classical MCT in outcome variables measuring treatment efficacy (positive symptoms, insight, self-esteem, and treatment adherence).
H2 - Exploratory (Platform validation): The ML-based predictive model will demonstrate significant predictive capacity to anticipate post-treatment response to MCT on positive symptoms (especially delusions), insight, self-esteem, and treatment adherence, as assessed by the AUC (Area Under the Curve).
H3 - Exploratory (Patient satisfaction): Patient satisfaction with P-MCT will be comparable or greater than with classical MCT, as assessed through both quantitative measures and qualitative insights from focus groups.
H4 - Exploratory (Platform improvement): The integration of multimodal comprehensive pre-post treatment data from the classical MCT group will refine the machine learning algorithm, resulting in improved predictive accuracy for future patient cohorts.

## 2 Methods and analysis

### 2.1 Study design

This is a randomized, double-blind, parallel-group, exploratory proof-of-concept trial designed to assess the superiority of P-MCT over classical MCT. Participants are recruited from five countries (Poland, Germany, France, Chile, and Spain) and are randomly assigned to one of the two treatment conditions. Participants will include in-patient and out-patient volunteers based on center availability. Following baseline assessment, participants are randomized to either the classical Metacognitive Training (MCT) or the Personalized MCT (P-MCT) condition using a computer-generated four-block randomization procedure generated by the technological team (UPC), ensuring allocation concealment. The experimental intervention will be P-MCT. Participants receiving classical MCT will serve as an active control group following the current program. The study will be double-blind, as patients and evaluators will not be aware of the assigned treatment condition. The trial has been registered in clinicaltrials.gov (ID: NCT06603922; https://www.clinicaltrials.gov/study/NCT06603922; 19/09/24), where any deviations from this protocol will also be recorded.

Both interventions will consist of 10 one-hour sessions, delivered in group format once or twice a week, depending on site feasibility. Some groups will be delivered MCT or P-MCT in online format to allow representative recruitment. Patient data will be fed into an ML platform prior to the intervention. The platform generates a personalized treatment response prediction to allow tailoring of the intervention by selecting individualized homework tasks targeting specific cognitive deficits. Allocation concealment during treatment will be ensured via sealed opaque envelopes prepared by an independent researcher not involved in recruitment or assessment. The platform does not store data, and thus predictions will be manually recorded for both treatment conditions. Only the P-MCT condition will receive personalization following the platform’s predictions, while the classical MCT predictions will be stored to analyze the platform’s accuracy at predicting the results of classical MCT. Post-treatment assessments will be conducted after the intervention and subsequently at a six-month follow-up evaluation. Full details of the study design are outlined in **Supplementary Figure S2**, and platform input and output information, and an example of the homework can be found in **Supplementary Figure S3**.

### 2.2 Participants

The sample will consist of patients with a diagnosis of non-affective psychosis, according to DSM-5 criteria (40). Participants will be recruited from multiple clinical centers (associated to the following clinical partners: Fundació Sant Joan de Déu [FSJD], Universitätsklinikum Hamburg-Eppendorf [UKE], Hôpitaux Universitaires de Strasbourg [UHS], Centre Hospitalier Universitaire de Nice (CHU de Nice), Institute of Psychology, Polish Academy of Science [PAoS] and Universidad del Valparaíso [UV]), including both inpatient and outpatient services, with a minimum of 51 patients per center. Recruitment will occur in psychiatric hospitals and community mental health centers. To facilitate recruitment, clinicians at these sites will receive detailed information about the study’s inclusion and exclusion criteria through local meetings and internal communications. Additionally, announcements will be posted in the centers to raise awareness and encourage referrals during routine clinical care. Inclusion criteria are (1): A DSM-5 diagnosis of non-affective psychosis (2); presence of positive symptoms during the past year (represented by a Positive and Negative Syndrome Scale [PANSS (41)] score equal or over 3 on the items measuring Delusions, Suspiciousness, or Grandiosity); (3) aged between 18 and 65 years old; (4) stable clinical condition with no expected changes in medication (as confirmed by clinical services); (5) no severe cognitive deficits (global assessment and/or information from clinical services). Exclusion criteria are: (1) previous attendance in MCT groups within the past year; (2) neurological disorders, history of head trauma, or premorbid IQ below 70 (based on medical reports and/or other sources); (3) a score equal or over 6 on the Hostility and Suspiciousness items of the PANSS Positive subscale; (4) aggressive behavior (reported by clinical services); (5) high suicide risk (verified through the DIAMOND clinical interview). Patients who do not meet the inclusion criteria will not be enrolled in the study but will be offered alternative treatment options or MCT at later stages.

The inclusion and exclusion criteria were designed to balance internal validity with real-world clinical representativeness. Criteria were primarily informed by those most employed across the studies that contributed to the development of the ML predictive platform, ensuring consistency between the training dataset and prospective trial population. The requirement for positive symptoms (PANSS ≥ 3) ensures the presence of clinically relevant MCT targets, while the upper threshold for hostility/suspiciousness (PANSS ≤ 6) maintains therapeutic dynamics and avoid group issues, while maintaining group representativeness and excluding severe cases and at the same time without overly restricting sample representativeness. The stable medication criterion reduces confounding from pharmacological changes, while cognitive and safety exclusions (IQ < 70, high suicide risk, aggressive behavior) ensure participants can meaningfully engage with the intervention. This approach avoids overly stringent criteria that would limit generalizability while maintaining sufficient homogeneity to detect treatment effects (42).

### 2.3 Measures

Data will be collected on sociodemographic, clinical, cognitive (neurocognitive, metacognitive, social-cognitive), and psychological variables. Given the transnational nature of the study, official validated translations of all instruments were used for each participating country. When validated translations existed but item ordering differed between countries, administration was standardized according to the official English version to ensure consistency across sites. For instruments lacking official translations in some specific languages (e.g., The Internal, Personal, and Situational Attributions Questionnaire (IPSAQ), Fish Task) and for consortium-developed scales (e.g., modified Patient-Reported Impact of Symptoms in Schizophrenia (PRISS) scale to monitor symptoms during treatment, satisfaction with MCT treatment), forward-backward translation procedures were conducted by independent bilingual researchers to ensure semantic and conceptual equivalence. The instruments used and their characteristics are presented in **Supplementary Table S1**.

Blood samples will be drawn from participants at baseline and post-treatment. In both centers collecting blood samples (FSJD and UV), leukocyte subpopulation counts obtained from blood count analyses and C-reactive protein levels will be collected. At the FSJD site, peripheral expression levels of SP transcription factors (SP1 and SP4) will be analyzed as part of a subproject aimed at identifying biomarkers with transcriptional reprogramming capacity involved in the response to MCT. Biomarkers are included because they provide objective biological indicators of inflammatory and transcriptional processes that may influence cognitive functioning and symptom severity in psychosis. These processes are increasingly recognized as moderators of treatment response in psychological interventions. Including biomarkers enables subgroup analyses to determine whether MCT efficacy varies by biological profile.

Incorporating biomarker data will refine the ML algorithm. This iterative process will determine which biomarkers consistently improve predictive accuracy, guiding future versions of the platform toward more biologically informed personalization.

Following the intervention, qualitative data on patient satisfaction will be collected through focus groups at each center, exploring participants’ experiences, perceived benefits, barriers, and overall satisfaction with the intervention. This data will complement the quantitative measures collected post-treatment and at follow-up.

### 2.4 Data collection

Pre-treatment, post-treatment, and follow-up assessments will be conducted by blinded, trained psychologists at each site. These evaluations will take place over the course of one to two interviews. Assessors will be trained in standardized administration of instruments to ensure equal collection across centers.

Nursing staff will be responsible for performing cubital vein blood counts for separation of peripheral blood mononuclear cells. Blood will be stored at 4°C until processed. Additionally, the transfer of samples to the Parc Sanitari Sant Joan de Déu (PSSJD) biobank will be requested following a prior agreement for participation in the study. The analyses will be conducted at the PSSJD Molecular Psychiatry Laboratory (SP1 and SP4 analysis) and the Hospital Sant Joan de Déu Clinical Laboratory (C-reactive protein and complete blood count). Blood will be collected from participants in the sites at UV and FSJD.

This study is in accordance with the World Medical Association’s Declaration of Helsinki and was approved by the Sant Joan de Déu Ethics Committee (Reference: PIC-110-24), the Institute of Psychology, Polish Academy of Science (Reference: IP.403.21.2024), Ethics Committee of Valparaíso San Antonio Health Service of Chile (Reference: N°48/2024), the local psychological ethics committee at the center for psychosocial medicine (LPEK) at the University Medical Center Hamburg-Eppendorf (Reference: LPEK-0796) and the Comité de protection des personnes Ile de France (Reference: 25.02907.000469). Since the study involves human participants, evaluations will be conducted once informed consent has been obtained. The personnel conducting the assessments, blood extractions, blood counts, and molecular analyses will be blind to the study condition assigned to each participant. All variables will be measured at baseline, post-treatment, and six months after completion, except for the DIAMOND and TEC scales, which will only be administered at baseline; the PRISS scale, which will be administered at the end of each session to monitor symptoms during treatment; patient satisfaction with treatment, which will be collected post-treatment and at follow-up; and the biological samples, which will be collected only within two weeks before the start of the intervention and within two weeks after its completion.

Data will be entered into a secure, password-protected database. Coding will follow standardized formats. Range checks and validation rules will be applied. Data will be stored at each site in compliance with GDPR and local regulations.

For qualitative data, each center will carry out two focus groups (one for the personalized MCT group and one for the classical MCT group), with 5–10 participants per group. Sessions will be audio-recorded, transcribed verbatim, anonymized, and translated into English. Field notes will also be kept. Monthly meetings with representatives from all centers will be held to triangulate results and ensure consistency across sites.

### 2.5 Interventions

Participants who meet inclusion criteria will be randomized into one of the two study conditions: (1) Metacognitive Training (MCT) and (2) Personalized Metacognitive Training (P-MCT). Sessions will run concurrently in all centers from October 2024 until April 2025.

MCT consists of 10 one-hour group sessions (3–10 participants). It includes ten modules referring to common cognitive issues and biases in solving problems in psychosis. MCT is effective in reducing symptoms and improving cognitive biases that are involved in the onset and maintenance of psychosis (12). The topics of MCT are the following: attribution blaming and taking credit (module 1), jumping to conclusions (modules 2 & 7), changing beliefs (module 3), deficits in theory of mind and other social cognition domains (modules 4 and 6), overconfidence in errors (module 5), mood (module 8), self-esteem (module 9) and self-stigma (module 10). Each session follows a protocol defined in the manual ‘Metacognitive Training for Psychosis’ (MCT). The format (online or in-person) and frequency (once or twice a week) will vary by center depending on staff resources; this information will be considered as covariables in the analysis. Participants receive worksheets at the end of each module to continue working on the modules at home.

The personalization strategy was developed through a two-day in-person consortium meeting dedicated to integrating ML predictions into treatment. Consistent with the dual objectives of personalizing treatment while maintaining accessibility, the consortium decided against full individualization of sessions, which would have reduced the number of patients able to access group-based MCT. Instead, P-MCT differs from classical MCT exclusively in homework content, while group sessions remained identical in both groups. Participants are asked to discuss their homework tasks with their MCT therapist exclusively to preserve blindness to treatment condition. After the follow-up assessment, participants are informed of which treatment group they were allocated to and the homework tasks given to the other group, as well as any extra personalized tasks not received are offered to them.

A key strategic decision concerned how to personalize homework. Rather than reinforcing domains predicted to improve most, the consortium adopted a recovery-oriented approach by targeting the three outcome domains with the lowest predicted improvement out of the five domains discussed to best represent successful treatment response (positive symptoms, insight, self-esteem, and treatment adherence). personalized exercises were developed by clinicians within the consortium using (1) clinical practice guidelines and (2) predictive features identified using SHAP values from the ML model (39), ensuring alignment between clinical relevance and data-driven indicators. Sixteen exercises (four per outcome domain) were developed and standardized in length and complexity to ensure that any differences in treatment effects arise from personalization rather than workload. Homework assignment follows a fixed and reproducible rule: for each participant, the platform identifies the three outcome domains with the lowest predicted improvement. Across the ten MCT sessions, homework is assigned by cycling repeatedly through these three domains (Domain 1 → Domain 2 → Domain 3 → repeat). Because the program includes ten sessions, Domain 1 receives four exercises, while Domains 2 and 3 each receive three exercises. Within each domain, exercises are delivered sequentially (Exercise 1 to Exercise 4), ensuring a consistent rotation structure tailored to the participant’s predicted needs while maintaining standardization of the overall intervention. The established order of tasks represents the degree of relevance of the related factor detected by the ML model. For instance, in the Positive Symptom analysis, related factors associated with improvement in positive symptoms included positive symptoms themselves, negative symptoms, JTC and self-reflection, in this order. As a result, in order to target positive symptoms, the four homework tasks related to this domain include (1) psychoeducation on positive symptoms, (2) an activity planner to reduce negative symptoms, (3) a task to identify and correct JTC biases, combining self-reflection with JTC improvement, and (4) positive symptoms psychoeducation on stress management.

Group sessions will be delivered by trained clinical psychologists or psychiatrists (https://clinical-neuropsychology.de/metacognitive-training-e-training/) with prior experience in MCT. All facilitators will undergo standardized training in both P-MCT and classical MCT, and monthly meetings will include supervision to ensure protocol fidelity. Data from all participants’ baseline assessment will be input into the platform, and its results will be used to personalize the homework for participants in the P-MCT condition only. Results from the MCT condition will be stored and only used to confirm the platform’s prediction accuracy.

In terms of adherence monitoring and ensuring protocol fidelity, participants are asked to complete a questionnaire after every session attended to record their symptomatology over the previous seven days, the completion of homework and their attendance to the session (session number and module attended). Other than the offered MCT sessions, participants will continue to receive their routine care through their usual center, including medication and any other non-MCT psychological treatment. Clinicians and MCT trainers will monitor for adverse events and discontinue the allocated intervention in case of worsening symptoms. Participants are allowed to drop out of the study at any point, and they are informed that they have the right to withdraw from their therapy group at any time when they sign the informed consent forms. Reasons for withdrawal, adherence to sessions and homework and reasons for missing data will be recorded.

### 2.6 Sample size calculation

To achieve a two-tailed size effect (Cohen’s *d*) of 0.5 with 95% power, using the total PANSS scores and balanced observations in the two groups, the estimated size is 210 in total. Considering 20% of loss to follow-up, we aim to include a total of 252 patients in the study.

### 2.7 Data analysis

#### 2.7.1 Comparative efficacy (H1)

To assess the interventions’ efficacy, improvements in the proximal targets of MCT, as defined in recent meta-analyses and meta-reviews (11, 12), will be analyzed using statistical methods (43). These targets include global positive symptoms, including delusions, cognitive insight, self-esteem and treatment adherence. Regression models for repeated measures will be used for each proximal target, with the treatment group (P-MCT or classical MCT) included as a covariate for comparison. Additionally, mean differences between baseline and post-treatment scores in each group will be compared using Student’s t-tests, or Mann-Whitney tests if the assumption of normality is violated. Missing data will be handled using multiple imputation techniques. Subgroup analyses will be conducted based on sex, age, and baseline symptom severity. Sensitivity analyses will assess robustness of findings. ANCOVAs will explore whether responses differ across participant profiles (44), with treatment as the independent variable and composite participant profiles as a fixed factor. Covariates will include number of sessions attended, online vs. face-to-face delivery, and session frequency. Participant profiles will be identified using unsupervised ML techniques.

#### 2.7.2 Platform validation (H2)

In the classical MCT group, the predictive accuracy of the platform will be evaluated by comparing actual results with predictions in the five key variables (positive symptoms, delusions, insight, self-esteem, and treatment completion), with predictive performance assessed using the AUC for classification models (45) and RMSE and the R^2^ determination coefficient for regression models (46). In the P-MCT group, the analysis will focus on whether this approach led to improved outcomes compared to baseline predictions. Effect sizes will be calculated using Cohen’s d.

#### 2.7.3 Patient satisfaction (H3)

Patient satisfaction will be assessed using a mixed-methods approach, combining quantitative measures collected post-treatment and at follow-up with qualitative data from focus groups conducted at each center. Focus groups will be transcribed verbatim, coded and analyzed using content analysis using ATLAS.ti, and grouped into categories and subcategories reflecting recurrent themes (satisfaction, perceived benefits, and barriers). Methodological rigor will follow Guba and Lincoln’s criteria of dependability, credibility, transferability, and confirmability (47). Triangulation across researchers and sites will be performed in monthly consortium meetings to enhance reliability and credibility. Representative quotes will be reported while maintaining anonymity.

#### 2.7.4 Platform improvement (H4)

Exploratory analyses using unsupervised ML techniques will examine whether integration of comprehensive pre-post distal treatment data, including quantitative clinical, cognitive, psychological measures and blood biomarker data (e.g., depression, trauma, cognitive bias, SP1/SP4 expression), can refine the ML platform and improve predictive accuracy for future patient cohorts. Qualitative data will not be used for platform improvement.

This combined approach allows for integration of quantitative outcomes with qualitative insights, providing a comprehensive assessment of intervention efficacy and patient experiences across the five participating countries.

## 3 Discussion

The present study constitutes a pioneering effort in the development of a platform aimed at optimizing psychosis treatment strategies by integrating a personalized approach centered on the individual characteristics of each patient. While existing literature supports the need to tailor interventions to the specific attributes of each individual, current clinical practice seldom implements this approach due, among other factors, to economic constraints and the limited scientific knowledge regarding the criteria that should guide treatment personalization.

MCT emerges as an ideal candidate for evaluating the feasibility of a personalized psychological treatment approach in psychosis for several reasons. Firstly, its efficacy has been extensively demonstrated both in the short and long term (12). Secondly, it is recommended in therapeutic guidelines as a first-line intervention for individuals with psychosis, addressing the psychological underpinnings of the disorder. Furthermore, MCT has shown positive outcomes across diverse psychosis subpopulations, suggesting its applicability to various diagnoses and disease stages. Moreover, its manualized format ensures interinstitutional comparability and facilitates implementation by mental health professionals with appropriate training. Additionally, its group-based application enhances cost-effectiveness compared to individual therapies.

The PERMEPSY project demonstrates a high degree of feasibility, supported by the consortium’s expertise in researching psychological interventions for psychosis, particularly MCT, as well as in the development of digital platforms and the application of machine learning. Prior to designing the randomized trial and the development of the predictive platform, the consortium conducted comprehensive systematic reviews of the literature to identify the most relevant variables associated with treatment response (11, 15, 19, 37, 38). These reviews, carried out by all partners according to their specific areas of expertise, ensured that the variables selected for inclusion in the retrospective harmonized database and the trial were both evidence-based and clinically meaningful. Variables identified by previous meta-analyses from researchers outside the consortium were also considered (12, 13, 48). This preliminary work strengthens the scientific foundation of the project and guarantees that the data collected are relevant for both the machine learning analysis and clinical decision-making. Moreover, the consortium has already collected and harmonized the necessary data for ML-based analyses and the creation of the platform prototype (39, 49, 50). The clinical team’s capacity to recruit patients in both past and ongoing studies further strengthens the feasibility of the trial.

Despite robust evidence confirming the efficacy of psychological interventions in symptom reduction and relapse prevention (51), only 10% to 25% of individuals with psychosis have access to such treatments (52). This is particularly concerning given that 50% to 75% of patients discontinue antipsychotic medication (5). The PERMEPSY initiative seeks to bridge this gap by providing a personalized psychological intervention tailored to patients’ specific needs. It is estimated that profiling patients and delivering individualized treatment could reduce relapse rates by over 70%, thereby helping patients regain a sense of purpose, enhancing their functional capacities, and increasing their overall recovery prospects. This approach is also expected to yield a significant reduction in both direct and indirect costs associated with psychosis.

Machine learning based technological solutions have the potential to enhance the identification of predictive variables for treatment response, thereby facilitating clinical decision-making. In the PERMEPSY study, a harmonized retrospective database was used to train a machine learning algorithm capable of predicting changes in key clinical and functional outcomes, such as positive symptoms, delusions, insight, self-esteem, between others. These predictions are generated based on composite patient profiles derived from pre-treatment data (39, 50). The platform thus supports clinicians to recognize objective problem areas, such as cognitive biases to address in the therapy. By combining the recognition of object problem areas from the ML algorithm with information about subjective concerns reported by patients, the MCT can be tailored to the individual needs. Beyond these clinical and cognitive predictors, this study also exploratorily examines whether blood biomarkers (inflammatory markers and SP transcription factors at selected sites) can contribute to refining predictive models of MCT response, offering preliminary insights into potential biological correlates of therapeutic change. Furthermore, incorporating a gender-sensitive approach in the early stages of treatment could further optimize outcomes. The development of the prototype platform and its implementation in clinical practice therefore represents a significant methodological advancement, enabling patient stratification based on data-driven insights and technology-assisted personalization (39).

While PERMEPSY constitutes an innovative initiative in applying personalized medicine principles to psychological interventions in psychosis, its implementation presents certain methodological challenges. The multicentric nature of the trial may introduce variability in MCT delivery, a factor that will be mitigated through a standardized training program, monthly supervision sessions, and staff mobility for trainers. Additionally, although the total sample size (*n* = 252) is sufficient to detect overall group differences, statistical power may be inadequate for conducting detailed subgroup analyses (e.g., by gender or comorbidities). To address this limitation, ML models prioritizing the most predictive variables will be employed, thereby reducing the risk of overfitting.

Finally, this study will also contribute to the development of knowledge regarding the ethical and legal aspects of patient stratification, as well as the regulatory frameworks for ML-based clinical decision support systems across various European Union countries and beyond. In fact, the consortium is already working on a regulatory roadmap in order to meet requirements for medical device certification, allowing the platform to be used in clinical settings. The findings may redefine mental health care standards by promoting more precise, cost-effective, and patient-centered interventions, in contrast to conventional therapeutic models. In line with this, the project provides an opportunity to adhere to current regulatory frameworks with the aim of facilitating the future translation of the developed platform into a certified medical device.

PERMEPSY represents a groundbreaking initiative in translating personalized medicine principles into the domain of psychological interventions for psychosis. By integrating advanced ML techniques within a transnational clinical trial, the project seeks not only to validate a predictive model but also to establish methodological foundations for future research in this emerging field. The potential benefits, ranging from resource optimization to reducing chronicity, justify the logistical and technical challenges inherent to its execution. The results of this trial could redefine mental health care standards, prioritizing precise, patient-centered interventions over outdated therapeutic paradigms.

## 4 Ethics and dissemination

Patient data included in the PERMEPSY database will be collected and stored in compliance with the Ethical standards established by the World Medical Association in the Declaration of Helsinki at its current version (75^th^) (53, 54). The study was approved by the Sant Joan de Déu Ethics Committee (Reference: PIC-110-24), the Institute of Psychology, Polish Academy of Science (Reference: IP.403.21.2024), Ethics Committee of Valparaíso San Antonio Health Service of Chile (Reference: 48/2024), the local psychological ethics committee at the center for psychosocial medicine (LPEK) at the University Medical Center Hamburg-Eppendorf (Reference: LPEK-0796) and the Comité de protection des personnes Ile de France (Reference: 25.02907.000469).

Patient safety will be monitored throughout the study. Case report forms (CRFs) at each site have been designed to systematically record adverse events voluntarily reported by participants at each assessment point. Additionally, a modified version of the Patient-Reported Impact of Symptoms in Schizophrenia (PRISS) scale will be administered after each MCT session to monitor symptom changes during treatment and identify potential adverse effects. Any serious adverse events will be immediately reported to the designated responsible physician at each center, who will implement appropriate medical measures according to local clinical protocols and regulatory requirements. Serious adverse events will also be reported to local ethics committees in accordance with institutional and national regulations.

Personal data will be codified and stored securely and confidentially, not leaving each center’s archives. Upon completion of data collection from all centers, patient identification of patients recruited outside one’s own center will be unavailable through pseudonymization. The final prospective database will be anonymized and will not contain any personal data from participants. The anonymized prospective database will be openly available 10 years after the end of the project from the Cora repository (https://cora.csuc.cat/en/rdr-research-data-repository/). Access to the anonymized final dataset will be granted to consortium partners. The dataset will be made publicly available 10 years after project completion. The length of stored data will be 10 years after the end of the project, and destruction of stored data in open access is 10 more years after the end of the embargo period (20 years after the end of the project).

All participants included in the study will be asked to give informed consent for their data to be used in future related projects, for their data to be anonymized and shared in open access after 10 years, and for their pseudonymized data to be shared with consortium partners during the project. An additional informed consent form will be collected to gather blood samples and store them in the PSSJD biobank. Patients will also be informed that their data will be destroyed at the 20-year mark. The treatment, communication and transfer of data of all participants will comply with Regulation EU 2016/679 of the European Parliament and of the Council of April 27, 2016, on the protection of natural persons with regard to the processing of personal data and the free movement of such data and with the Organic Law 3/2018, of December 5th, 2018, on the Protection of Personal Data and guarantee of digital rights. The justifying legal basis of data processing is the patient’s signed consent, in accordance with the provisions of article 9 of EU Regulation 2016/679.

The project’s outcomes will be communicated in expert scientific conferences, peer-reviewed publications in scientific journals (in open access where applicable) and in community events or as plain-language summaries to reach participants and other lived experienced individuals, healthcare professionals, the public, and other relevant stakeholders. Trial outcomes will be registered and updated in public trial registries. This study protocol and all future publications will adhere to the Consolidated Standards of Reporting Trials (CONSORT) guidelines for transparent reporting of randomized controlled trials. The Standard Protocol Items: Recommendations for Interventional Trials (SPIRIT) checklist (55) can be found in the **Supplementary Table S4**. The project’s past, present and future dissemination actions can be viewed from the project’s website: permepsy.org.
