# Supplementary material for: PERMEPSY: a multicentre, randomized, double-blind proof-of-concept trial of personalized metacognitive training for adults with psychosis — a study protocol
Source: Front Psychiatry. 2026 Mar 6;17:1711659. doi: 10.3389/fpsyt.2026.1711659 (PMC13003757; doi:10.3389/fpsyt.2026.1711659)
Supplement: Supplementary file 1 [file DataSheet1.pdf]

SUPPLEMENTARY MATERIAL

Table S1. Instruments used in the prospective clinical trial.

INSTRUMENTS	OUTCOMES	RESPONSE RANGE	HYPOTHESES
**Clinical variables**			
**DIAMOND** (Tolin *et al.*, 2018)	Diagnosis, presence of substance dependence, presence of suicide risk	Confirmation of the presence or absence of diagnostic criteria according to the DSM-5.	H4
**PANSS: The Positive and Negative Syndrome Scale**<br>(Kay, Fiszbein and Opler, 1987)	Psychotic symptoms	General Symptomatology Subscale: Scores range from 16 to 112.<br><br>Positive and Negative Symptomatology Scales: Scores range from 7 to 49.	H1-2: Positive scale<br><br>H4: Negative and General scales
**PSYRATS: The Psychotic Symptom Rating Scale**<br>(Haddock *et al.*, 1999)	Psychotic symptoms	Hallucination Subscale: Score range from 0 to 44.<br><br>Delusion Subscale: Scores range from 0 to 24.	H1-2
**PHQ-9: The Patient Health Questionnaire-9**<br>(Kroenke, Spitzer and Williams, 2001)	Depression	The score ranges from 0 to 27. A higher score indicates greater severity.	H4
**RSES: The Rosenberg's Self-Esteem Scale**<br>(Rosenberg, 2011)	Self-Esteem	Scores range from 10 to 40. Higher scores indicate better self-esteem.	H1-2
**SUMD**: **The Scale to Assess Unawareness of Mental Disorder**<br>(Amador *et al.*, 1993)	Clinical insight	Scores range from 0 to 15. Higher scores indicate a poorer awareness or attribution.	H1-2
**PRISS: Patient-Reported Impact of Symptoms in Schizophrenia**<br>(Moreno-Küstner *et al.*, 2022)	Psychotic Symptoms	Scores range from 14 to 70. Higher scores indicate greater impact of psychotic symptoms.	H1-2: number of sessions attended<br><br>H4: other measures
**Social cognition**			

**IPSAQ: The Internal, Personal, and Situational Attributions Questionnaire** (Kinderman and Bentall, 1996)	Cognitive biases in psychosis	Scores range from 0-100% in 4 items. Higher percentages in each item represent an attributional style tendency.	H4
**The Hinting Task**<br>(Corcoran, Mercer and Frith, 1995)	Theory of mind	Scores range from 0 to 6. Lower scores indicate poorer performance. Three stories are evaluated at each time point.	H4
**BCFT**: **Baron Cohen's Face Test**<br>(Baron-Cohen *et al.*, 1997)	Emotion Processing	Confidence scores range from 4 to 20. Accuracy scores range from 0 to 4. Higher scores indicate better performance in emotional processing.	H4
**Metacognition**			
**Fishes task, Jumping To Conclusions Task**<br>(Moritz *et al.*, 2010; Speechley, Whitman and Woodward, 2010)	Jumping to conclusions (JTC) bias	Draws to decision: Scores from 1 to 2 indicate the presence of JTC bias, while scores above 2 indicate the absence of JTC bias. Scores range from 1 to 10.	H4
**BCIS: The Beck Cognitive Insight Scale**<br>(Beck, 2004)	Cognitive insight	Self-certainty: As the score increases, it indicates greater confidence in one's interpretations, within a range of 0 to 18.<br>Self-reflection: A higher score reflects a greater inclination to question one's own beliefs, within a range of 0 to 27.<br>Composite Index: Scores range from -27 to 45.	H1-H2
**CBQ-P: The Cognitive Biases Questionnaire for Psychosis** (Peters *et al.*, 2014)	Cognitive biases in psychosis	Scores range from 30 to 90. Higher scores indicate a greater presence of cognitive biases.	H4
**RFQ**: **The Reflective Functioning Questionnaire**<br>(Fonagy *et al.*, 2016)	Reflective functioning (mentalisation)	Scores range from 0 to 24. Higher scores indicate difficulties in mentalisation. Some items are reverse-coded, and responses to	H4

		items are recoded as 0, 1, 2 or 3.	
**Predisposing factors**			
**TEC: The Traumatic Experience Checklist** (Nijenhuis, Van Der Hart and Kruger, 2002)	Traumatic events	Scores range from 0 to 95. Higher scores indicate greater exposure to traumatic experiences.	H4
**QSLE-SV: The Short Version of the Questionnaire of Stressful Life Events** (Butjosa *et al.*, 2023)	Stressful life events	Scores range from 0 to 9. Higher scores indicate higher exposure to stressful experiences.	H4
**BHS: The Short Version of the Beck Hopelessness Scale** (Perczel Forintos *et al.*, 2013)	Hopelessness	Scores range from 0 to 4. Higher scores indicate higher hopelessness.	H4
**Cognition**			
**TMT: The Trail-Making Test** (Reitan and Wolfson, 1995)	Visual scanning and working memory	Test scored in time taken to complete task. Higher scores indicate worse performance.	H4
**Psychosocial Functioning**			
**GAF: Global Assessment of Functioning** (adapted by Hall, 1995 (Hall, 1995), from Global Assessment Scale [GAS] by Endicott et al., 1976 (Endicott, 1976))	General functioning – clinical and social functioning	The score range spans from 1 = severely affected to 100 = superior functioning across a broad range of activities. There are two sub-scores reflecting clinical and social functioning.	H4
**Quality of life**			
**WHOQOL-BREF** (The Whoqol Group, 1998)	Quality of life	Mental Health Domain: Scores range from 7 to 35.<br>Psychological Health Domain: Score range from 6 to 30.<br>Social Relationships Domain: Score range from	H4

		3 to 15.<br>Environmental Health Domain: Scores range from 8 to 40.	
**ISI: Insomnia Severity Index** (Bastien, 2001)	Insomnia	Scores range from 0 to 28:<br>- 0-7: Absence of insomnia<br>- 8-14: Mild insomnia<br>- 15-21: Moderate insomnia<br>- 22-28: Severe insomnia	H4
**Psychological outcomes**			
**ISMI-10: Brief version of the Internalized Stigma of Mental Illness Scale**<br>(Ritsher, Otilingam and Grajales, 2003; Boyd, Otilingam and DeForge, 2014)	Subjective experience of stigma	Scores range from 4 to 10. Higher scores indicate greater internalized stigma.	H4
**B-IRI: Brief Form of the Interpersonal Reactivity Index**<br>(Ingoglia, Lo Coco and Albiero, 2016)	Empathy	It is divided into 4 subscales that are evaluated independently: Perspective Taking, Fantasy, Empathic Concern and Personal Distress<br>Scores range from 4 to 20 in each subscale.<br>Higher scores on each subscale indicate higher levels of that specific aspect of empathy.	H4
**Satisfaction**			
**Satisfaction with MCT treatment** (Moritz and Woodward, 2007)	Satisfaction	Scores range from 5 to 50. Higher scores indicate greater satisfaction with the training.	H3

Figure S2. Flowchart of the study.

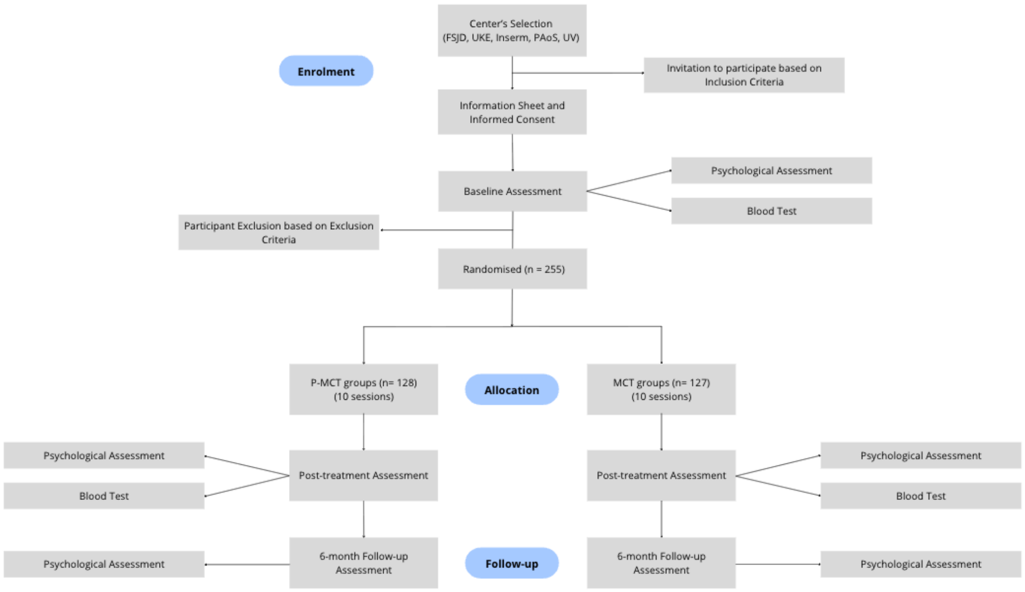

Figure S3. A) Platform input, B) output variables and C) personalised homework example.

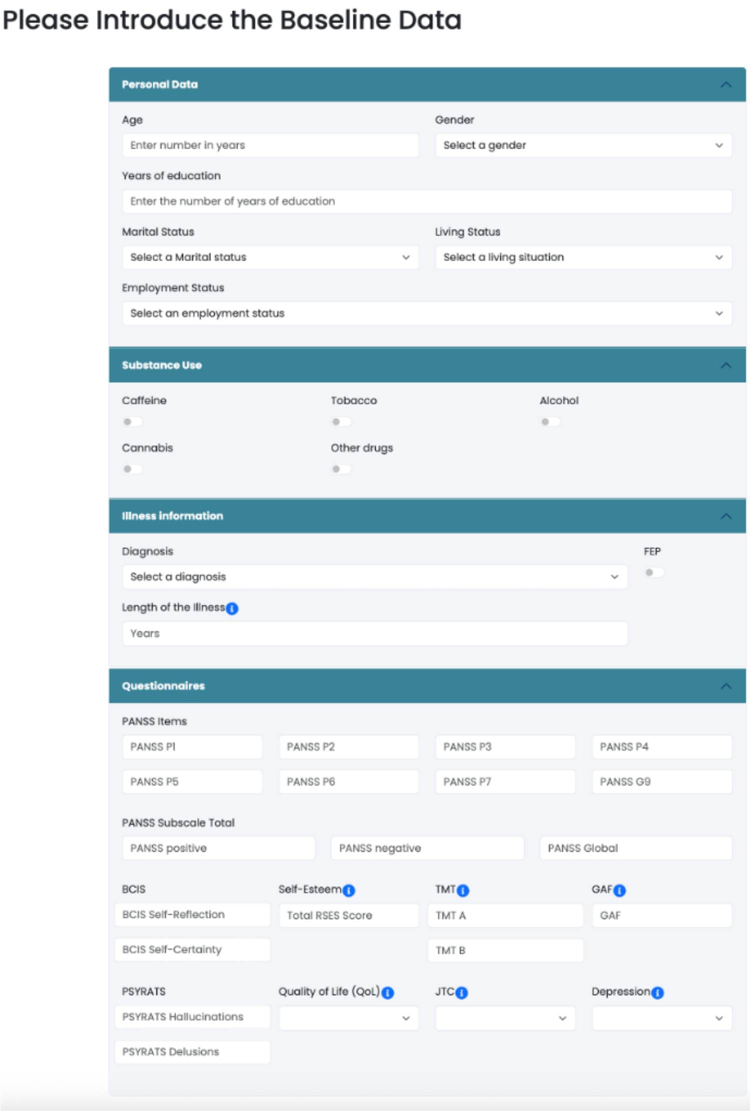

A)

B)

Questionnaires	Initial Assessment	Predictions	Percentage
PANSS Positive:	49	23	62.91%
Delusions according to PANSS:	21	10	63.15%
PSYRATS Hallucination:	11	4	10.71%
PSYRATS Delusions:	13	8	20.37%
RSES total:	12	19	24.57%
BCIS composite index:	-12	5	-27.74%
BCIS Self-Reflection:	12	14	6.18%
BCIS Self-Certainty:	24	10	60.08%
MCT Completion:		Yes	

Download CSV   Download PDF   Show model versions used

We inform you that these predictions regarding the effectiveness of MCT therapy have been generated using Artificial Intelligence technology. The system does not meet the requirements of the Artificial Intelligence Act of the European Union and the European Medical Device Regulation as the current Technology Readiness Level is 3 (Experimental proof of concept). You can consult the explanation of the predictions at this EU AI Act.

Percentage difference

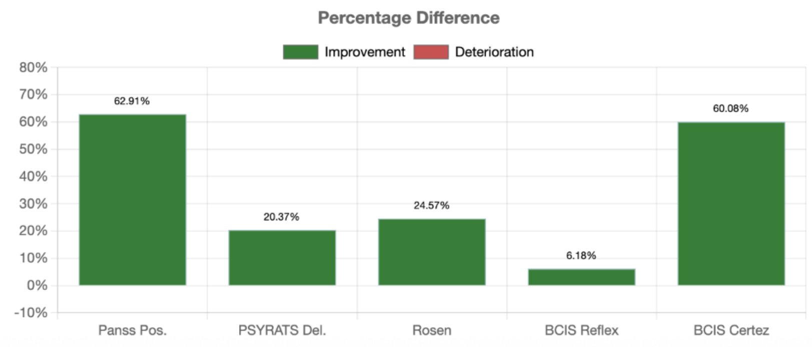

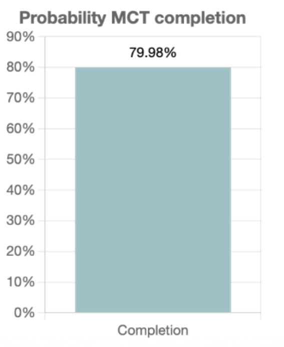

Note: FEP = First Episode Psychosis; PANSS = Positive and Negative Syndrome Scale; PX= Item number X from the Positive subscale of the PANSS; G9 = Item number 9 of the General subscale of the PANSS; BCIS = Beck Cognitive Insight Scale; RSES= Rosenberg Self-Esteem Scale; TMT = Trail Making Test; GAF = Global Assessment of Functioning; PSYRATS = Psychotic Symptom Rating Scale; JTC = Jumping to Conclusions; MCT = Metacognitive Training.

C)

What are positive symptoms?

Positive symptoms are one of the possible symptoms of schizophrenia**.** They include **delusions and/or hallucinations**, which are exaggerated ideas, perceptions, or actions that often make it difficult to distinguish between what is real and what is not.

Hallucinations can affect all the senses

- **Auditory:** Hearing voices commenting on my actions (or communicating with each other); hearing sounds others can't hear.
- **Olfactory:** smelling unusual odours that no one else notices.
- **Tactile:** feeling sensations of being touched, a physical presence or phenomenon inside my body (e.g., feeling something under my skin).
- **Visual:** seeing images or things (more or less clearly) others don't see.
- **Gustatory:** Tasting unusual or persistent flavours that no one else notices.

Delusions can relate to many themes:

- **Persecution**: feeling attacked, harassed, spied on or the victim of a conspiracy (e.g., feeling pursued by the mafia, government or illuminati).
- **Reference**: believing objects or situations send personal messages (e.g., TV host sending me hidden messages).
- **Grandeur**: believing in possessing extraordinary skills or knowledge (e.g., feeling like a rock star whose talent is unrecognized, or destined to save the world).
- **Mystical**: having exaggerated beliefs about supernatural, spiritual or religious things (e.g. receiving prophetic messages from Buddha in dreams).
- **Erotomaniac**: believing someone is in love with me, without any obvious signs (e.g. convinced a singing star I've never met is secretly in love with me).
- **Guilt**: believing I've done something terrible and unforgivable without evidence (e.g. being thinking a car accident was caused by my thoughts).
- **Thought theft/disclosure**: fearing my thoughts are stolen or reused without my knowledge (e.g. thinking my memory lapses are due to thoughts theft).
- **Influence**: feeling that my sensations, impulses, thoughts or actions are controlled by an external force (e.g. someone using a voodoo doll to control me).

Let's try to spot the positive symptoms!

For the past three days, Tom has noticed a black car with tinted windows parked along his route to work. He finds it suspicious and wonders if someone is hiding inside, watching his movements. When he asks his friend Paul about it, Paul assures him that cars with tinted windows are becoming more common. However, upon arriving at his office, Tom locks himself in and puts his cell phone on airplane mode to ensure no one can spy on him.

Question 1: Do you think that Tom is having hallucinations and/or delusions in this story (Yes or No)? _____________

Question 2: If yes, what type of symptoms (hallucinations or delusions)? ______________

Question 3: If Tom is having delusions in this story, what type are they (Persecution, Reference, Grandeur, Mystical, Erotomaniac, Guilt, Thought theft/disclosure, Influence)?

____

Table S4. SPIRIT 2025 checklist of items to address in a randomized trial protocol.

Section / Topic	No	SPIRIT 2025 checklist item description	Reported on page no.
**Administrative information**			
Title and structured summary	1a	Title stating the trial design, population, and interventions, with identification as a protocol	1
	1b	Structured summary of trial design and methods, including items from the World Health Organization Trial Registration Data Set	1-2
Protocol version	2	Version date and identifier	Footer
Roles and responsibilities	3a	Names, affiliations, and roles of protocol contributors	1, 2, 21
	3b	Name and contact information for the trial sponsor	21
	3c	Role of trial sponsor and funders in design, conduct, analysis, and reporting of trial; including any authority over these activities	21
	3d	Composition, roles, and responsibilities of the coordinating site, steering committee, endpoint adjudication committee, data management team, and other individuals or groups overseeing the trial, if applicable	19-20
**Open science**			
Trial registration	4	Name of trial registry, identifying number (with URL), and date of registration. If not yet registered, name of intended registry	2, 7
Protocol and statistical analysis plan	5	Where the trial protocol and statistical analysis plan can be accessed	2, 7
Data sharing	6	Where and how the individual de-identified participant data (including data dictionary), statistical code, and any other materials will be accessible	18-19
Funding and conflicts of interest	7a	Sources of funding and other support (e.g., supply of drugs)	21
	7b	Financial and other conflicts of interest for principal investigators and steering committee members	21
Dissemination policy	8	Plans to communicate trial results to participants, healthcare professionals, the public, and other relevant groups (e.g., reporting in trial registry, plain language summary, publication)	19
**Introduction**			
Background and rationale	9a	Scientific background and rationale, including summary of relevant studies (published and unpublished) examining benefits and harms for each intervention	3-5

	9b	Explanation for choice of comparator	5, 11-13
Objectives	10	Specific objectives related to benefits and harms	5-6
**Methods: Patient and public involvement, trial design**			
Patient and public involvement	11	Details of, or plans for, patient or public involvement in the design, conduct, and reporting of the trial	7-8, 9-11
Trial design	12	Description of trial design including type of trial (e.g., parallel group, crossover), allocation ratio, and framework (e.g., superiority, equivalence, non-inferiority, exploratory)	6-7
**Methods: Participants, interventions, and outcomes**			
Trial setting	13	Settings (e.g., community, hospital) and locations (e.g., countries, sites) where the trial will be conducted	7-8
Eligibility criteria	14a	Eligibility criteria for participants	8
	14b	If applicable, eligibility criteria for sites and for individuals who will deliver the interventions (e.g., surgeons, physiotherapists)	9-10
Intervention and comparator	15a	Intervention and comparator with sufficient details to allow replication including how, when, and by whom they will be administered. If relevant, where additional materials describing the intervention and comparator (e.g., intervention manual) can be accessed	3, 11-13
	15b	Criteria for discontinuing or modifying allocated intervention/comparator for a trial participant (e.g., drug dose change in response to harms, participant request, or improving/worsening disease)	13
	15c	Strategies to improve adherence to intervention/comparator protocols, if applicable, and any procedures for monitoring adherence (e.g., drug tablet return, sessions attended)	13
	15d	Concomitant care that is permitted or prohibited during the trial	13
Outcomes	16	Primary and secondary outcomes, including the specific measurement variable (e.g., systolic blood pressure), analysis metric (e.g., change from baseline, final value, time to event), method of aggregation (e.g., median, proportion), and time point for each outcome	8-9, 27-30 (Supplementary)
Harms	17	How harms are defined and will be assessed (e.g., systematically, non-systematically)	13,18
Participant timeline	18	Time schedule of enrollment, interventions (including any run-ins and washouts), assessments, and visits for participants. A schematic diagram is highly recommended (see Figure)	11

Sample size	19	How sample size was determined, including all assumptions supporting the sample size calculation	13
Recruitment	20	Strategies for achieving adequate participant enrollment to reach target sample size	7-8
**Methods: Assignment of interventions**			
Randomization:			
Sequence generation	21a	Who will generate the random allocation sequence and the method used	6
	21b	Type of randomization (simple or restricted) and details of any factors for stratification. To reduce predictability of a random sequence, other details of any planned restriction (e.g., blocking) should be provided in a separate document that is unavailable to those who enroll participants or assign interventions	6
Allocation concealment mechanism	22	Mechanism used to implement the random allocation sequence (e.g., central computer/telephone; sequentially numbered, opaque, sealed containers), describing any steps to conceal the sequence until interventions are assigned	6
Implementation	23	Whether the personnel who will enroll and those who will assign participants to the interventions will have access to the random allocation sequence	6
Blinding	24a	Who will be blinded after assignment to interventions (e.g., participants, care providers, outcome assessors, data analysts)	6-7, 9-10
	24b	If blinded, how blinding will be achieved and description of the similarity of interventions	6-7, 11
	24c	If blinded, circumstances under which unblinding is permissible, and procedure for revealing a participant's allocated intervention during the trial	11
**Methods: Data collection, management, and analysis**			
Data collection methods	25a	Plans for assessment and collection of trial data, including any related processes to promote data quality (e.g., duplicate measurements, training of assessors) and a description of trial instruments (e.g., questionnaires, laboratory tests) along with their reliability and validity, if known. Reference to where data collection forms can be accessed, if not in the protocol	8-11
	25b	Plans to promote participant retention and complete follow-up, including list of any outcome data to be collected for participants who discontinue or deviate from intervention protocols	13
Data management	26	Plans for data entry, coding, security, and storage, including any related processes to promote data quality (e.g., double data entry; range checks for data values).	10

		Reference to where details of data management procedures can be accessed, if not in the protocol	
Statistical methods	27a	Statistical methods used to compare groups for primary and secondary outcomes, including harms	13-15
	27b	Definition of who will be included in each analysis (e.g., all randomized participants), and in which group	13-15
	27c	How missing data will be handled in the analysis	13
	27d	Methods for any additional analyses (e.g., subgroup and sensitivity analyses)	13-15
**Methods: Monitoring**			
Data monitoring committee	28a	Composition of data monitoring committee (DMC); summary of its role and reporting structure; statement of whether it is independent from the sponsor and funder; conflicts of interest and reference to where further details about its charter can be found, if not in the protocol. Alternatively, an explanation of why a DMC is not needed	18
	28b	Explanation of any interim analyses and stopping guidelines, including who will have access to these interim results and make the final decision to terminate the trial	13-15
Trial monitoring	29	Frequency and procedures for monitoring trial conduct. If there is no monitoring, give explanation	13
**Ethics**			
Research ethics approval	30	Plans for seeking research ethics committee/institutional review board approval	10
Protocol amendments	31	Plans for communicating important protocol modifications to relevant parties	7
Consent or assent	32a	Who will obtain informed consent or assent from potential trial participants or authorized proxies, and how	19
	32b	Additional consent provisions for collection and use of participant data and biological specimens in ancillary studies, if applicable	19
Confidentiality	33	How personal information about potential and enrolled participants will be collected, shared, and maintained in order to protect confidentiality before, during, and after the trial	18-19
Ancillary and post-trial care	34	Provisions, if any, for ancillary and post-trial care, and for compensation to those who suffer harm from trial participation	13

Supplementary Material S5. Reference list for used instruments.

Amador, X.F. *et al.* (1993) ‘Assessment of insight in psychosis’, *The American Journal of Psychiatry*, 150(6), pp. 873–879. Available at: https://doi.org/10.1176/ajp.150.6.873.

Baron-Cohen, S. *et al.* (1997) ‘Another Advanced Test of Theory of Mind: Evidence from Very High Functioning Adults with Autism or Asperger Syndrome’, *Journal of Child Psychology and Psychiatry*, 38(7), pp. 813–822. Available at: https://doi.org/10.1111/j.1469-7610.1997.tb01599.x.

Bastien, C. (2001) ‘Validation of the Insomnia Severity Index as an outcome measure for insomnia research’, *Sleep Medicine*, 2(4), pp. 297–307. Available at: https://doi.org/10.1016/S1389-9457(00)00065-4.

Beck, A. (2004) ‘A new instrument for measuring insight: the Beck Cognitive Insight Scale’, *Schizophrenia Research*, 68(2–3), pp. 319–329. Available at: https://doi.org/10.1016/S0920-9964(03)00189-0.

Boyd, J.E., Otilingam, P.G. and DeForge, B.R. (2014) ‘Brief version of the Internalized Stigma of Mental Illness (ISMI) scale: Psychometric properties and relationship to depression, self esteem, recovery orientation, empowerment, and perceived devaluation and discrimination.’, *Psychiatric Rehabilitation Journal*, 37(1), pp. 17–23. Available at: https://doi.org/10.1037/prj0000035.

Butjosa, A. *et al.* (2023) ‘Development and validation of a short version of the questionnaire of stressful life events (QSLE)’, *Clinical Psychology & Psychotherapy*, 30(6), pp. 1464–1470. Available at: https://doi.org/10.1002/cpp.2886.

Corcoran, R., Mercer, G. and Frith, C.D. (1995) ‘Schizophrenia, symptomatology and social inference: Investigating “theory of mind” in people with schizophrenia’, *Schizophrenia Research*, 17(1), pp. 5–13. Available at: https://doi.org/10.1016/0920-9964(95)00024-G.

Endicott, J. (1976) ‘The Global Assessment Scale: A Procedure for Measuring Overall Severity of Psychiatric Disturbance’, *Archives of General Psychiatry*, 33(6), p. 766. Available at: https://doi.org/10.1001/archpsyc.1976.01770060086012.

Fonagy, P. *et al.* (2016) ‘Development and Validation of a Self-Report Measure of Mentalizing: The Reflective Functioning Questionnaire’, *PLOS ONE*. Edited by K. Laws, 11(7), p. e0158678. Available at: https://doi.org/10.1371/journal.pone.0158678.

Haddock, G. *et al.* (1999) ‘Scales to measure dimensions of hallucinations and delusions: the psychotic symptom rating scales (PSYRATS)’, *Psychological Medicine*, 29(4), pp. 879–889. Available at: https://doi.org/10.1017/s0033291799008661.

Hall, R.C.W. (1995) ‘Global Assessment of Functioning’, *Psychosomatics*, 36(3), pp. 267–275. Available at: https://doi.org/10.1016/S0033-3182(95)71666-8.

Ingoglia, S., Lo Coco, A. and Albiero, P. (2016) ‘Development of a Brief Form of the Interpersonal Reactivity Index (B–IRI)’, *Journal of Personality Assessment*, 98(5), pp. 461–471. Available at: https://doi.org/10.1080/00223891.2016.1149858.

Kay, S.R., Fiszbein, A. and Opler, L.A. (1987) ‘The positive and negative syndrome scale (PANSS) for schizophrenia’, *Schizophrenia Bulletin*, 13(2), pp. 261–276. Available at: https://doi.org/10.1093/schbul/13.2.261.

Kinderman, P. and Bentall, R.P. (1996) ‘A new measure of causal locus: the internal, personal and situational attributions questionnaire’, *Personality and Individual Differences*, 20(2), pp. 261–264. Available at: https://doi.org/10.1016/0191-8869(95)00186-7.

Kroenke, K., Spitzer, R.L. and Williams, J.B.W. (2001) ‘The PHQ-9: Validity of a brief depression severity measure’, *Journal of General Internal Medicine*, 16(9), pp. 606–613. Available at: https://doi.org/10.1046/j.1525-1497.2001.016009606.x.

Moreno-Küstner, B. *et al.* (2022) ‘Patient-reported impact of symptoms in schizophrenia scale (PRISS): Development and validation’, *Acta Psychiatrica Scandinavica*, 145(6), pp. 640–655. Available at: https://doi.org/10.1111/acps.13417.

Moritz, S. *et al.* (2010) ‘Different sides of the same coin? Intercorrelations of cognitive biases in schizophrenia’, *Cognitive Neuropsychiatry*, 15(4), pp. 406–421. Available at: https://doi.org/10.1080/13546800903399993.

Moritz, S. and Woodward, T.S. (2007) ‘Metacognitive training in schizophrenia: from basic research to knowledge translation and intervention’, *Current Opinion in Psychiatry*, 20(6), pp. 619–625. Available at: https://doi.org/10.1097/YCO.0b013e3282f0b8ed.

Nijenhuis, E.R.S., Van Der Hart, O. and Kruger, K. (2002) ‘The psychometric characteristics of the traumatic experiences checklist (TEC): first findings among psychiatric outpatients’, *Clinical Psychology & Psychotherapy*, 9(3), pp. 200–210. Available at: https://doi.org/10.1002/cpp.332.

Perczel Forintos, D. *et al.* (2013) ‘Proposal for a Short Version of the Beck Hopelessness Scale Based on a National Representative Survey in Hungary’, *Community Mental Health Journal*, 49(6), pp. 822–830. Available at: https://doi.org/10.1007/s10597-013-9619-1.

Peters, E.R. *et al.* (2014) ‘Cognitive Biases Questionnaire for Psychosis’, *Schizophrenia Bulletin*, 40(2), pp. 300–313. Available at: https://doi.org/10.1093/schbul/sbs199.

Reitan, R.M. and Wolfson, D. (1995) ‘Category test and trail making test as measures of frontal lobe functions’, *The Clinical Neuropsychologist*, 9(1), pp. 50–56. Available at: https://doi.org/10.1080/13854049508402057.

Ritsher, J.B., Otilingam, P.G. and Grajales, M. (2003) ‘Internalized stigma of mental illness: psychometric properties of a new measure’, *Psychiatry Research*, 121(1), pp. 31–49. Available at: https://doi.org/10.1016/j.psychres.2003.08.008.

Rosenberg, M. (2011) ‘Rosenberg Self-Esteem Scale’. American Psychological Association. Available at: https://doi.org/10.1037/t01038-000.

Speechley, W.J., Whitman, J.C. and Woodward, T.S. (2010) ‘The contribution of hypersalience to the “jumping to conclusions” bias associated with delusions in schizophrenia’, *Journal of Psychiatry and Neuroscience*, 35(1), pp. 7–17. Available at: https://doi.org/10.1503/jpn.090025.

The Whoqol Group (1998) ‘The World Health Organization quality of life assessment (WHOQOL): Development and general psychometric properties’, *Social Science & Medicine*, 46(12), pp. 1569–1585. Available at: https://doi.org/10.1016/S0277-9536(98)00009-4.

Tolin, D.F. *et al.* (2018) ‘Psychometric Properties of a Structured Diagnostic Interview for *DSM-5* Anxiety, Mood, and Obsessive-Compulsive and Related Disorders’, *Assessment*, 25(1), pp. 3–13. Available at: https://doi.org/10.1177/1073191116638410.
